# Guanfacine in the Treatment of a Child Diagnosed with Tourette Syndrome: A Case Report

**DOI:** 10.1192/j.eurpsy.2024.1451

**Published:** 2024-08-27

**Authors:** L. T. Durán Sandoval, M. D. C. Blasco Fresco, E. de la Fuente Ruiz

**Affiliations:** ^1^Psychiatry, Hospital Universitario Miguel Servet; ^2^Departamento de Medicina, Psiquiatría y Dermatología, Universidad Zaragoza, Zaragoza, Spain

## Abstract

**Introduction:**

Tourette syndrome (TS) is a neurodevelopmental disorder characterized by the development of persistent and changing motor and phonic tics over time. The presence of at least two motor tics and one vocal tic that have persisted for at least a period of 1 year is required, and which developed before the age of 18. The most commonly used pharmacological treatment are antipsychotics, with a preference for atypical antipsychotics such as aripiprazole or risperidone. Clonidine and guanfacine have shown effectiveness in suppressing tics, and although generally less effective than antipsychotics, some authors are considering them as first-line treatments. The treatment is also influenced by any comorbidities the patient may present.

**Objectives:**

To enumerate in a clinical case the pharmacological alternatives for TS, which vary according to the patient’s comorbidities and the intensity of the tic symptoms.

**Methods:**

Case study. Anamnesis of the patient and their family.

**Results:**

A 12-year-old boy presenting simple motor and vocal tics for over a year. At the same time that a valuation is requested by child psychiatry, the mother also requests follow-up by neuropediatrics. Other causes are ruled out, an EEG is performed, and a TS diagnosis is made. The initial treatment was low-dose aripiprazole with partial effectiveness. After 3 months, he presents an exacerbation of the tics, interfering with his social and academic life, making it impossible to attend classes. The mother takes him to emergency services, and he is admitted to pediatrics. During the stay in pediatrics, he is diagnosed with Attention Deficit Hyperactivity Disorder, in addition to confirming the TS diagnosis. Extended-release methylphenidate is initiated (neuropediatrics). After starting methylphenidate, the patient’s tics worsen, also presenting insomnia and hyporexia. Due to the diagnosis of ADHD, school failure, and affective symptoms (hypothymia), atomoxetine is initiated. The tics become constant and incapacitating. As the dose of aripiprazole is increased, the child presents extrapyramidal effects. As a therapeutic alternative, guanfacine is initiated, progressively discontinuing aripiprazole. Currently, the child is stable from motor and vocal tics, allowing him to lead a normalized life.

**Conclusions:**

Although guanfacine is not as effective in reducing tics as antipsychotics, since the latter produce more side effects, it is justifiable to use it. This drug is capable of enhancing the therapeutic effect and reducing the adverse effects that antipsychotics could produce. Guanfacine may be a good alternative as a first line in the treatment of Tourette Syndrome with or without attention deficit disorder and hyperactivity .

**Disclosure of Interest:**

None Declared

